# Value of Tc-99m DMSA renal scan in assessing the prognosis and outcome of acute renal failure due to sepsis

**DOI:** 10.1186/cc12355

**Published:** 2013-03-19

**Authors:** S Fawzi, K Mashour, A Amin, H Gamal

**Affiliations:** 1 Cairo University, Cairo - Manial, Egypt

## Introduction

Acute renal failure (ARF) is a common complication in patients admitted to the ICU. Sepsis is also a well-known risk factor for the development of ARF. The combination of ARF and severe sepsis was reported to carry a mortality up to 70% whereas the mortality of ARF alone is 40 to 45%. The aim of the study is to evaluate the role of renal perfusion scanning in detecting the prognosis and outcome of patients with acute renal failure due to sepsis.

## Methods

Forty patients with acute renal failure due to sepsis, aged between 15 and 74 years, were enrolled in the study. They were monitored for their ICU prognosis and outcome after doing renal perfusion scanning. All patients were subjected to routine ICU and laboratory investigations including APACHE II and SOFA score.

## Results

Thirty patients had normal renal scan and 10 patients had abnormal renal scan. The mortality percentage was higher among abnormal renal scan cases (three out of 10, 30%) compared with cases with normal renal scan (seven out of 30, 23.3%) with nonsignificant *P *value: 0.6. The median length of stay/day in ICU was longer among nonsurvivors than survivors 15.5 ± 10, 11.5 ± 8, *P *value: 0.058 (approaching significance). APACHE II score was higher in nonsurvivors than survivors 23.9 ± 3.2, 19.6 ± 4.2, *P *value: 0.0001. The percentage of mortality among cases that needed mechanical ventilation was higher (nine out of 16, 56.3%) compared with mortality cases that did not need mechanical ventilation (one out of 24, 4.2% with *P *value: 0.0001).

## Conclusion

ARF may exert an independent adverse effect on outcome in septic and septic shock patients. It is also a risk factor for mortality. Tc-99m DMSA scanning is useful for detecting renal dysfunction and help to predict the outcome and prognosis.
